# Soil Microplastic Pollution and Microbial Breeding Techniques for Green Degradation: A Review

**DOI:** 10.3390/microorganisms12061147

**Published:** 2024-06-05

**Authors:** Zhuang Xiong, Yunfeng Zhang, Xiaodie Chen, Ajia Sha, Wenqi Xiao, Yingyong Luo, Jialiang Han, Qiang Li

**Affiliations:** Key Laboratory of Coarse Cereal Processing, Ministry of Agriculture and Rural Affairs, Sichuan Engineering & Technology Research Center of Coarse Cereal Industrialization, School of Food and Biological Engineering, Chengdu University, No. 2025, Chengluo Avenue, Longquanyi District, Chengdu 610106, China; xiongzhuang2000@126.com (Z.X.); zhangyunfeng@cdu.edu.cn (Y.Z.); cxd0512@126.com (X.C.); shaajia19980108@126.com (A.S.); xwq990713@126.com (W.X.); lyy1478963@126.com (Y.L.)

**Keywords:** microplastics, microplastic-degrading microorganisms, strain breeding, biodegradation, soil pollution

## Abstract

Microplastics (MPs), found in many places around the world, are thought to be more detrimental than other forms of plastics. At present, physical, chemical, and biological methods are being used to break down MPs. Compared with physical and chemical methods, biodegradation methods have been extensively studied by scholars because of their advantages of greenness and sustainability. There have been numerous reports in recent years summarizing the microorganisms capable of degrading MPs. However, there is a noticeable absence of a systematic summary on the technology for breeding strains that can degrade MPs. This paper summarizes the strain-breeding technology of MP-degrading strains for the first time in a systematic way, which provides a new idea for the breeding of efficient MP-degrading strains. Meanwhile, potential techniques for breeding bacteria that can degrade MPs are proposed, providing a new direction for selecting and breeding MP-degrading bacteria in the future. In addition, this paper reviews the sources and pollution status of soil MPs, discusses the current challenges related to the biodegradation of MPs, and emphasizes the safety of MP biodegradation.

## 1. Introduction

Agriculture and industrial growth are key aspects of human civilization [1]. During the agricultural production process, plastics are mainly used in mulch films, as well as packaging materials for fertilizers, hormones, and pesticides, etc. [2]. The extensive use of these plastic items can result in a buildup of plastics in the soil. As they are stable and difficult to break down, they will remain in the ground for an extended period of time [3,4,5]. Exposure to weather, UV radiation, and biodegradation for an extended period can lead to physical, chemical, and biological changes in plastics, ultimately breaking them down into microplastics (MPs) that are less than 5 mm in diameter [6,7,8]. MPs are a significant source of plastic pollution in soil ecosystems and agroecosystems [2,9,10]. The global environmental challenge of soil pollution caused by MPs needs to be closely addressed. 

When MPs linger in the soil, they can lead to pollution in the ecosystem [11]. Much research has demonstrated that MPs can penetrate plants through their root systems, resulting in negative impacts on root traits, development, and nutrient uptake [12]. Liu et al. [13] demonstrated that MPs are able to enter the rice root system and translocate into the stem and leaf parts, thus potentially translocating up through the food chain. This enrichment in the food chain eventually reaches humans. Several studies have shown that different kinds of MPs have been found in human feces [14,15,16], the placenta [17], and breast milk [18], indicating that MPs have entered the human body and that they are being delivered to the whole body through circulation [19]. If MPs are left in the body, they can cause damage such as oxidative stress, inflammation, metabolic disorders, and potentially birth defects [20,21]. 

Taking into account the severe harm caused by MPs, the problem of MP pollution needs to be addressed as soon as possible [22]. MPs are typically degraded using physical [23,24,25], chemical [26], and biological methods [27]. Traditional physical and chemical methods, including incineration, photodegradation, and electrochemical oxidation, have been used for many years [28]. However, these techniques are time-consuming, expensive, and may even lead to secondary pollution [29,30,31,32,33]. Therefore, they are not sustainable remediation methods. Compared to traditional physical and chemical methods, the biodegradation method has the advantages of no secondary pollution, high efficiency, and environmental protection. Biodegradation can take advantage of the natural ability of microorganisms to degrade MPs in soils. These microorganisms participate in metabolic pathways by producing specific enzymes to completely degrade or convert MPs into less harmful products [34]. 

Although the biodegradation of MPs shows good prospects, its practical application is limited. The reasons are mostly due to the low adaptability of the degrading bacteria, a lack of competitiveness, a long degradation time, a poor effect, and a low utilization rate of the target pollutants [35,36]. By utilizing suitable strain-breeding technology, the efficiency of MP-degradation can be enhanced, thus facilitating the widespread adoption of biodegradation [37]. Therefore, finding methods that can effectively improve the degradation efficiency of MP-degrading bacteria has become the focus of current research. Strain-breeding technology, as a key link in microbial degradation, is of great significance in screening strains that have the ability to degrade MPs efficiently. Strain-breeding technology is a method of improving microbial traits through artificial intervention and plays a crucial role in the breeding of MP-degrading bacteria. As technology continues to advance, there has been an increase in the exploration of microbial resources, leading to the discovery of strains capable of efficiently degrading MPs. These strains have the natural ability to degrade MPs, and their degradation ability can be improved by strain-breeding techniques.

MP biodegradation is a hot topic in the field of environmental protection and is also one of the most challenging research directions. Currently, researchers around the world are striving to select and breed microorganisms that can efficiently and continuously degrade MPs. Although reports have summarized the microorganisms that can degrade different types of MPs [27], there is a lack of systematic summarization of the use of strain-breeding techniques to obtain MP-degradation strains. The latest progress in the breeding technology of degrading bacteria is very important for obtaining efficient degrading bacteria. Against this background, in this paper, we summarize for the first time the techniques of strain breeding for bacteria that degrade MPs. This paper provides the first systematic summary of strain-breeding techniques for MP-degrading bacteria to gain a better comprehension of the current progress, to recognize the challenges, and to forecast the future potential, as well as to provide an outlook on how to obtain efficient degrading bacteria. This paper is composed of four sections. In Section 1, the sources of MPs are reviewed to help reduce the possibility of MPs entering the soil environment at their source. The Section 2 delves into the present state and detrimental effects of MP pollution, shedding light on the seriousness of this pollution. In Section 3, the breeding techniques and application status of MP-degrading bacteria are systematically introduced. After this, Section 4 analyzes the challenges of the current research work and proposes potential solutions based on our findings. In summary, this study provides new ideas for the generation of efficient MP-degrading bacteria, which takes the bioremediation of MPs in soil a step further and helps accelerate the global realization of MP control.

## 2. Literature Retrieval

**Figure 1:**
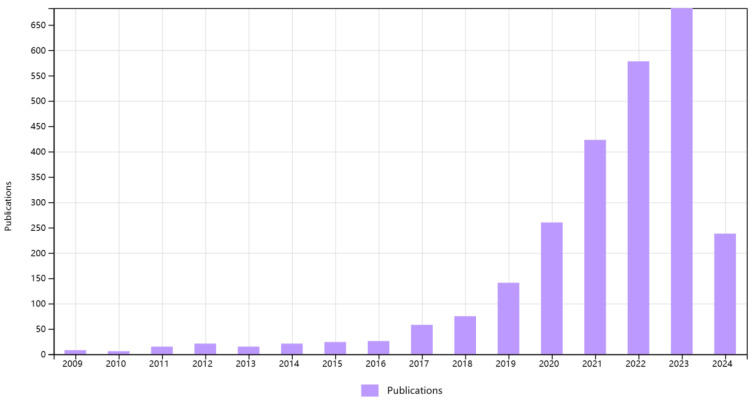
Figure of changes in the number of publications on MP pollution and MP degradation from 2009 to 2024.

In May 2024, we conducted a literature review to search for literature on MP pollution and MP degradation from the Web of Science database (Figure 1). The scientific literature search used search strings related to “Soil Microplastic Pollution”, “Microplastic Degradation”, and “Microbial Breeding Techniques”. We retrieved a total of 2592 related publications. A search through ScienceDirect with the keyword “Biodegradation of MPs in Soil” found approximately 1700 literature sources. This indicates that many researchers have carried out work on the biodegradation of MPs. However, reviews summarizing the application of microbial-breeding techniques in soil MP degradation are really scarce. Only 17 references were retrieved from the Web of Science under the theme “Microbial Breeding and Microplastics”. The earliest publication on this topic dates back to 2009. The number of studies on this topic has increased over time, reaching a peak in 2023 with a total of 683 publications related to this topic. Of these, the studies on the sources and pollution of MPs are experiments conducted under natural environmental conditions, while the experiments on the degradation of MPs are experiments conducted under laboratory conditions.

## 3. MP Contamination in Soil

### 3.1. Sources of MPs in Soil

**Figure 2:**
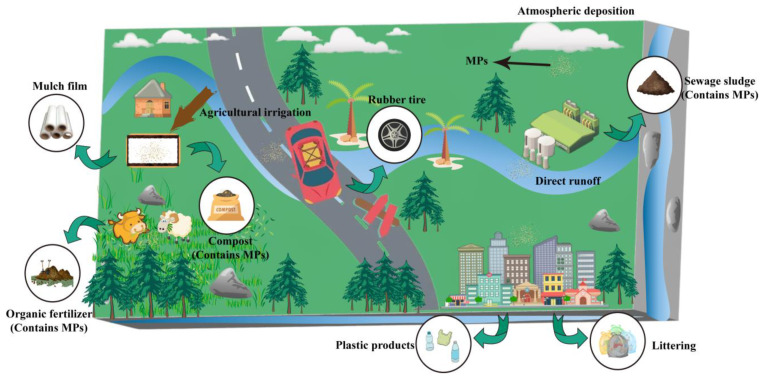
Major source of MPs in soils. Green arrows represent sources of MPs.

So far, there has not been a comprehensive field survey program to assess the distribution of MPs in soil. Studies have shown that soil contamination from MPs is a global issue [38]. The soil is seen as a significant reservoir of MP pollution, both absorbing and releasing it [39]. MPs in polluted soil environments primarily come from agricultural film residues, agricultural irrigation wastewater, organic fertilizers, sludge from wastewater treatment plants, seepage from plastic landfills, waste from plastic products, atmospheric sedimentation, random dumping, improper garbage disposal, and tire wear during car operations [40,41,42]. Figure 2 shows the main sources of MPs.

The sources of MPs are extensive and complex. Today, pollution from MPs has far exceeded people’s imagination. The detrimental effects of MPs must not be overlooked, and taking action against the issue of MP contamination is crucial. Therefore, clarifying the origin of MPs in soil is helpful in decreasing the production of MPs at their source.

#### 3.1.1. Agricultural Films

Agricultural film is mainly made of polyethylene (PE) and polyvinyl chloride (PVC), which have different thicknesses and colors. PE film is lightweight and has high light transmittance, while PVC film provides good heat insulation but has poor light transmittance and releases toxic and hazardous substances when burned [43]. The anticipated rise in global agricultural film utilization is expected to be 5.7%, attributed to the significant advocacy and application of film-mulching cultivation techniques [44]. Plastic covers are a major contributor to MP pollution in agroecosystems [45,46]. Due to the thinness of plastic mulch (approximately 8 to 50 mm), it is difficult to recycle plastic mulch from the soil after crops are harvested [47]. It is time-consuming and laborious to remove all the mulch films in farmland, so mulch films or their debris are often left in farmland [48,49]. The plastic films that remain on the farmland surface will cause photodegradation and become fragile under direct UV radiation [50,51,52]. Plastics that are already in a delicate state may be further broken up due to plowing and other soil tillage processes taking place in agricultural fields [53]. Zhou et al. [54] noted that in comparison to crop soils without plastic film, those covered with plastic film contained much higher amounts of MPs, averaging 571.2 pieces kg^−1^, while the uncovered crop soils had an average of 262.7 pieces kg^−1^. Additionally, the abundance of MPs increased with continuous plastic coverage. According to Huang et al. [50], the concentrations of MPs found in soils covered with plastic films for 5 to 24 years increased from 80.3 ± 49.3 pieces kg^−1^ to 1075.6 ± 346.8 pieces kg^−1^, respectively. The problem of elevated levels of MPs in soils is a result of the extensive use of agricultural films globally. This poses a greater risk of increasing the abundance of MPs in soils, which cannot be ignored.

#### 3.1.2. Organic Fertilizer

Organic fertilizers are widely used in soils nowadays, comprising animal and bird manures, crop straws, plant ashes, and sludges [55]. Organic fertilizer is an important input in agricultural production and is widely used in vegetable fields. Nevertheless, organic fertilizer is an important source of soil MPs [56]. With the improvement of organic fertilizer production technology, the particle size of MPs in organic fertilizer is becoming increasingly smaller. As a result, it is usually difficult to detect or identify them. Yang et al. [57] researched the amount of MPs in soil that had been exposed to the prolonged use of pig manure. Results indicated that the application of pig manure led to a notable rise in the amount of MPs in soil, with an average accumulation rate of 3.50 ± 1.71 million grains per hectare. Germany is one of the countries that has the strictest requirements on fertilizer quality in the world. A survey conducted by German researchers found that the abundance of MPs in organic fertilizers was found to be 14–895 particles kg^−1^ [58]. It was also discovered that MPs with a particle size of over 0.5 mm had a content range of 2.38 to 180 milligrams per kilogram [59]. However, the statistical data mentioned above for organic fertilizers still do not include MPs below 0.5 mm. Australia and Germany have formulated relevant standards for MP content in organic fertilizers. Australia allows 0.5% of hard plastics and 0.05% of light plastics in organic fertilizers, while Germany allows 0.1% of plastics in organic fertilizers [58]. The use of organic fertilizers has become a significant pathway for MPs to enter the soil. Hence, nations globally should contemplate creating suitable laws and policies to minimize the potential of MPs from organic fertilizers seeping into the soil.

#### 3.1.3. Sludge

It is estimated that MPs discharged into agricultural soils through sewage sludge are among the largest sources [60]. Sludge application on agricultural land has increased the abundance of MPs in the soil, making a significant contribution [61]. Sludge is a fertilizer commonly used on farmland and is composed of organic material, trace elements, and essential nutrients like nitrogen, phosphorus, and potassium [59,62]. Approximately half of the cities in North America and Europe use sludge as fertilizer, and in countries such as Finland, the proportion of sludge used as fertilizer reaches 80% [2]. Despite this, evidence indicates that sludge contains a high number of MPs, with levels ranging between 1500 and 56,000 particles per kilogram [63,64,65,66]. During a survey of the wastewater treatment facilities along the Clyde River, it was discovered that the influent had a concentration of MPs at 15.7 ± 5.23/L. However, after passing through the treatment facilities, the effluent’s MP concentration dropped to 0.25 ± 0.04/L, resulting in a removal rate of 98% or more [64,67]. In addition, it is worth noting that the removed MPs are not degraded but hidden in the sludge. Consequently, when sludge is released into the soil, it will cause a buildup of MPs in the soil [68]. Removing MPs through conventional sludge pretreatment methods is a challenging task. In the EU, the annual usage of 4 × 10^6^ − 5 × 10^6^ t (dry weight) of sludge for composting on arable land is accompanied by the entry of approximately 4 × 10^5^ t of MPs into the soil [2,69,70]. In Australia, the total amount of MPs accumulated annually due to sludge application can reach 2.8 × 10^3^ − 1.9 × 10^4^ t [71]. Several studies have shown that sludge application to soil is associated with elevated levels of MPs, usually increasing with the number of applications [72,73,74,75]. Research has discovered that sludge includes hazardous elements like heavy metals, pathogens, antibiotics, parasite eggs, and persistent organic compounds. These elements can bond with MPs and worsen the soil pollution problem. Currently, there are few relevant research results available. Consensus is still lacking on how much MP contamination is present in soil [76].

#### 3.1.4. Surface Runoff and Agricultural Irrigation

MPs will enter farmland soil through surface runoff, infiltration, and agricultural irrigation. Piñon-Colin et al. [77] revealed that in the Tijuana region of Mexico, the concentration of MPs in stormwater runoff ranged from 66 to 191 particles L^−1^, from which the annual discharge of MPs from stormwater runoff was calculated to be about 8 × 10^5^–3 × 10^6^ particles hm^−2^, and this portion of the runoff can infiltrate or directly enter into agricultural soils. Globally, agricultural irrigation takes water from various sources, including surface water, groundwater, and reclaimed wastewater. Studies have found that MPs are found in large surface waters in China, such as the Changjiang River and Fanyang Lake [78,79,80]. The concentration of MPs in the Changjiang waters from Chongqing to Yichang ranged from 46.7 to 204 items L^−1^. In addition, the surface waters of Poyang Lake contained 5–34 items L^−1^, while the rivers on the Qinghai-Tibet Plateau had a content of 483–967 items m^−3^ [81]. The highest level of MP contamination in the groundwater of Illinois, USA, was recorded at 15.2 particles L^−1^ [82]. MPs become a source of MPs in the soil by hiding in water and entering the soil through runoff or irrigation.

#### 3.1.5. Atmospheric Deposition

MPs are also heavily introduced to the soil environment through atmospheric deposition [83]. The MPs released through atmospheric deposition are usually small in size. Allen et al. [84] found that the transport distance of MPs through the atmosphere can reach up to 95 km, and the quantity is astonishing. Dris et al. [85] studied the number of MPs in the atmosphere near Paris. The measurements showed that the daily number of MPs deposited in the atmosphere was 2–355 particles m^−2^, and the annual amount of fibrous MPs deposited in this area via the atmosphere was 3–10 t, most of which were fibrous. Of these, particles with sizes exceeding 1000 μm made up half of the total amount. Examining the composition of atmospheric fallout, researchers determined that natural fibers accounted for 50%, processed natural fibers for 21%, man-made plastic fibers for 17%, and a combination of both man-made and natural fibers for 12% [85]. Without proper disposal, domestic plastic waste can end up settling into nearby farmland through atmospheric precipitation, whether it is dumped directly or indirectly. Under the comprehensive action of various external factors, some plastic waste is weathered, broken, and degraded into smaller particles, releasing plastic residual debris into the soil environment through atmospheric deposition. Along the arterial highway, the wear of vehicle rubber tires generates MP particles. Kim et al. [86] pointed out that these rubber particles from tire wear can be transported to the soil near the highway through atmospheric precipitation or surface runoff. Yukioka et al. [87] reported that the abundance of MPs (size ranging from 0.01 to 0.05 mm) in the road dust of Kusatsu, Japan, was 2.0 ± 1.6 pieces m^−2^; in Kathmandu, Nepal, it was 12.5 ± 10.1 pieces m^−2^. Plastic particles emitted during building construction processes, plastic particles released by leakage or diffusion in industrial production, and dust released from old furniture also enter the soil environment through atmospheric deposition processes. For a more thorough understanding of MPs in the atmosphere, it is essential to investigate their spatiotemporal distribution, atmospheric transport characteristics, and the factors that affect them. This will allow for the estimation of their transport range and the flux of their sedimentation in atmospheric deposition, as well as the determination of their sources in the atmosphere.

### 3.2. Contamination Status of MPs in Soil

Soil pollution, which is caused by MPs, is widely regarded as a significant danger to ecosystems on a global scale. It has been observed to affect both abiotic and biotic elements [88,89,90,91,92,93]. MPs in the soil not only change its physicochemical properties [94,95] but also affect the growth and development of plants and animals in the soil [96,97,98,99,100]. Therefore, it is necessary to strengthen the study of the pollution and ecological hazards caused by MPs in soil.

Currently, the majority of research on MP contamination is centered on the ocean, with fewer studies conducted on land, particularly in agricultural soils. Differences in the mass and quantity units used to quantify the abundance of MPs in related studies make it challenging to compare the results, as there is a lack of uniformity in the units used. In this paper, studies by relevant researchers on the global MP pollution status in soils in recent years were analyzed, and the abundance and distribution characteristics of MPs in soils were summarized (Table 1). As shown in Table 1, soils in different countries and regions have been polluted by varying levels of MPs. This pollution status varies according to the time zone. MPs of a particle size smaller than 5 mm made up the majority found in soil. The soil in industrial areas contains a higher amount of MPs due to the deposition of small particles and debris during production or processing processes. In a Sydney, Australia, industrial area, thirteen soil samples were gathered, and the presence of MPs was detected to be between 300 and 67,500 mg kg^−1^ [101]. MP concentrations up to 55.5 mg kg^−1^ were found in 29 samples of floodplain soil in Switzerland, which is far lower than the concentration in the contaminated soil in Australia [102]. The significant disparity in MP content between the two locations stemmed from the fact that the sampling point in Australia was located in an industrial area with relatively severe pollution, while the sampling point in Switzerland was in a floodplain with relatively light pollution. 

The global soils contaminated with MPs are mainly farmland soil, sludge-irrigated soil, and film-planted soil. MPs are more abundant in sludge-irrigated soil but less so in film-planted soil. The average proportion of MP sizes 0–1 mm and 1–2 mm in vegetable fields in suburban Beijing was 43.0% and 35.1%, respectively, while the remaining 21.9% of MP sizes were distributed in 2–5 mm, with the quantities varying from 160 to 5520 items kg^−1^ [103]. The average concentration of MPs in the Mexican vegetable field soil reached 870 particles kg^−1^ [104]. In Southern Germany, due to the use of traditional tillage, farmland was less polluted by MPs. The abundance range was 0–1.25 microplastic particles (MPPs) per kilogram, and the average was 0.34 ± 0.36 MPPs per kilogram [53]. Compared with Germany, the concentration of MPs in the farmland soils of Canada, Iran, and Switzerland was higher, with the highest peak at approximately 600 particles kg^−1^ [75,102,105]. The abundance ranges of MPs in the farmland soils of Spain and Pakistan were similar [106,107]. The concentration of MPs in Chilean farmland soils was quite different, with values in the range of 600 to 10,400 particles kg^−1^ [108]. The Danish farmland soil contained the highest amount of MPs, reaching 236,000 no. kg^−1^ [109]. The concentration of MPs in farmland soils in Yunnan Province, China, is generally higher than in other areas. This may be related to the use of sludge composting and wastewater to irrigate farmland in Yunnan. In addition, Yunnan is rich in tourism resources, and the arrival of a large number of tourists will heighten the potential for soil contamination with MPs [110]. Agricultural mulch pollution and greenhouse planting are also among the reasons for the high abundance of MPs. Polymers such as PS and PE have been detected in the soils of greenhouses in the planting soils of Republic of Korea in the forms of debris, film, and fibers, with abundances of 10–7630 items kg^−1^ [111]. Samples were collected from a depth of 0–3 cm in the greenhouse planting soil in Wuhan, and the amount of MPs in the samples was notably greater than that in the vegetable plot at the same depth, with a total of 780 ± 129 items kg^−1^ [112]. The MP content in soil in Shaanxi was generally lower than that in agricultural mulch-contaminated vegetable plots. The MP concentration in soil was 1430–3410 items kg^−1^, and the MP concentration in agricultural mulch-contaminated vegetable plots was 6 × 10^5^ items kg^−1^ [113]. Moreover, as the years of mulch-film mulching increased, the concentration of MPs in the soil contaminated with agricultural mulch also rose. In Xinjiang, when the mulch-film mulching period was extended from five to thirty years, the MP content in the soil rose from 91.2 to 308.5 mg kg^−1^ [51].

**Figure 3:**
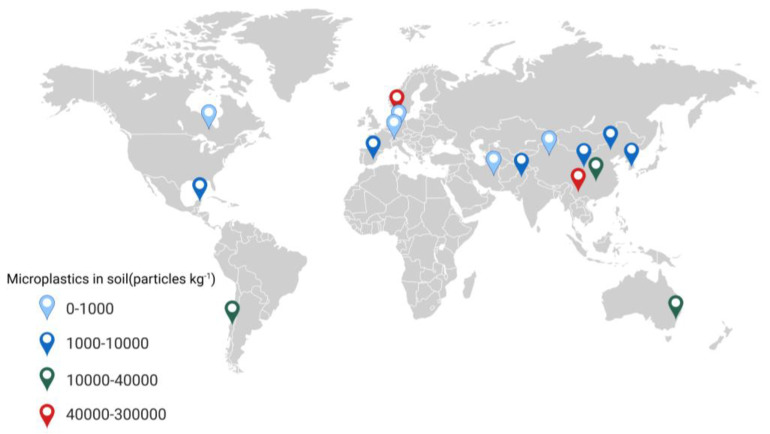
The abundance of MPs in soils in global typical areas (based on Table 1). Different colored pointers represent different abundances of MPs. Created with BioRender.com.

Table 1 shows that the spatial distribution of MPs is uneven. This could be associated with the degree of development, the population density, and the physical geographic characteristics of the country or region. Additional research is necessary in order to comprehend the factors that impact the dissemination and prevalence of MPs. Currently, research on the distribution of MPs in soil ecosystems is still in its early stages. Standardized methods for the analysis of MPs in soil need to be established, and a unified unit of MP abundance should be established for the comparison of different study results. For example, to express MPs in terms of quantity, the unit particles per kilogram (particles kg^−1^) may be used, and in terms of weight, the unit milligrams per kilogram (mg kg^−1^) may be used. The harmonized units will allow researchers to better compare the concentration of MPs in soils from different regions. Figure 3 displays the amount of MPs present in the soil of certain typical areas.

## 4. Strain-Breeding Technology

**Figure 4:**
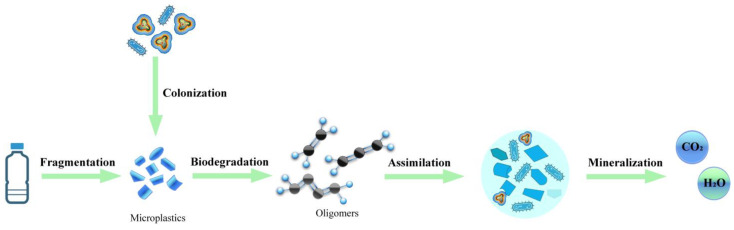
Microbial degradation process of MPs. Microorganisms first colonize the surface of the polymer, forming a biofilm, where the physical, chemical, and mechanical energy of the polymer changes. Biodegradation then takes place, where microbial activity breaks down the polymer into oligomers and monomers. This is followed by assimilation, where microbes obtain carbon sources, energy, and nutrients from the fragmented polymer. Eventually, the microbes mineralize the plastic, converting it to carbon dioxide, water, and other substances. Created with BioRender.com.

MPs can be eliminated through various physical, chemical, and biological methods [114]. Physical methods include incineration [115], ultraviolet (UV) radiation [116], and photocatalysis [117]. UV radiation and photocatalysis can also cause environmental pollution [118]. Incineration causes the emission of hazardous gases into the atmosphere, resulting in a polluted environment. At the same time, the use of chemical methods to remove MPs can leave chemical agents in the soil, which can harm human health and the natural world [119,120]. Biodegradation methods using bacteria and fungi can remove MPs without destroying the surrounding environment and biological communities. Biodegradation is typically considered a modification of organic compounds [114]. The biodegradation process of polymers includes three steps of microbial activity [121]. Eventually, they convert the plastic into carbon dioxide, water, and other substances [122]. In Figure 4, the degradation of MPs by microorganisms is depicted. Firstly, polymers undergo prolonged physicochemical reactions in which their carbon and hydrogen bonds are broken, generating free radicals and forming smaller molecules. Moreover, oxidation sites, such as hydroxyl and carboxyl groups, are formed on the surface of these polymers. This leads to changes in the surface morphology and structure of the polymers, enhancing their hydrophilicity and affinity for microorganisms, which promotes the biodegradation of the polymers [123]. Biodegradation is the following step, a process that consists of breaking down polymers into oligomers and monomers using microbial activities. The final step is assimilation, during which the microorganisms obtain a carbon source, energy, and nutrients from the broken polymer. Eventually, they convert the plastic into carbon dioxide, water, and other substances [122]. Although biodegradation is considered a green and sustainable technology for the degradation of MPs, the efficiency of MP removal using biological methods is usually low [124]. Biodegradation has not been widely utilized. By using appropriate strain-breeding technology, strains that can efficiently degrade MPs can be obtained, thus improving the efficiency of bioremediation for practical application.

With the continuous improvement and perfection of biotechnology, microbial-breeding technology has been widely used in all walks of life, especially in the screening of MP-degrading bacteria in soil. This has greatly improved the efficiency and quality of MP degradation. Microbial-strain-breeding technology is a breeding technology that uses genetic principles and technologies to transform a strain with a specific application purpose, to remove its adverse characteristics, retain beneficial traits, and improve its efficiency. Breeding at the microbial level is primarily based on natural mutations, from which strains with excellent traits are selected. These traits are then fixed in the genes through the process of selection. This includes natural selection breeding and mutagenesis breeding. Natural selection and breeding involve the selection of microorganisms during the reproduction process under normal growth and environmental conditions. This process eliminates mutations that are unfavorable for their survival and development, while retaining beneficial mutations for the semi-conservative replication of their own DNA. Mutation breeding is a technology that uses physical or chemical methods to artificially induce mutations in microorganisms and then selects excellent strains with target traits. Table 2 lists the MP-degrading strains obtained using different strain-breeding techniques.

### 4.1. Natural Breeding

After the invention of pure cultures of microorganisms, the natural cultivation of pure microorganisms has emerged. Natural selection is based on the spontaneous gene mutation of microorganisms or the breeding of strains containing desired traits to obtain excellent strains with target traits.

Currently, the use of natural breeding to obtain strains with the ability to degrade MPs is the most common method. Screening for MP-degraders through natural breeding focuses on selecting strains that can effectively utilize the carbon source of MPs and demonstrate strong degradation effects. Many scholars have successfully screened strains with MP degradation ability from aquatic ecosystems through natural breeding methods. For example, Harshvardhan and Jha [127] screened three strains out of sixty marine bacterial strains isolated from seawater along the coast of the Arabian Sea for their ability to grow in a medium with PE as the sole source of carbon. After 30 days, the maximum weight loss of PE was 1.75% for *Bacillus subtilis* H1584. Sun et al. [125] isolated 13 bacterial strains that can degrade MPs from Bohai Bay. Among the 13 isolated strains, *Exiguobacterium* a-1 showed potential for degrading polypropylene (PP), as it was able to degrade 9.20% of the additive-free PP film in 80 days. Additionally, Sarkhel et al. [128] isolated the bacterial strain *Vibrio* sp. PD6 and the fungal strain *Aspergillus* sp. from saline water. The degradation ability of a plastic-bottle polymer film is better, and over a period of 6 weeks, the bacterial strains degraded the polymer samples more than the fungal strains (35% for the bacterial strains and 22% for the fungal strains). Compared to the studies mentioned above, Nanthini Devi et al. [126] used the natural breeding method to screen for MP-degrading bacteria with higher efficiency. They isolated four strains from the Vaigai River in Madurai, India, and classified them as MPs (PE, PP) to be used as carbon sources for degradation experiments. After 21 days, when treated alone, the degradation rates of PP and PE were the highest among *Bacillus paramycoides* and *Bacillus cereus*; the degradation rates were 78.99% ± 0.005% and 63.08% ± 0.009%, respectively. During the joint treatment, the effects of *Bacillus cereus* and *Bacillus paramycoides* on PP and PE showed the highest degradation rates; the degradation rates were 78.62% ± 2.16% and 72.50% ± 20.53%, respectively. In addition, the generation of new functional groups confirmed that the strains had formed biofilms on the surface of the MPs. As the biofilm grows, the surface of the MPs cracks and breaks down, and changes in the functional groups indicate the process by which the MPs are degraded, proving their efficiency in degrading them.

More bacteria with MP-degradation capacity were screened from soil using natural colonization methods than from aquatic ecosystems. Auta et al. [130] selected two strains of bacteria out of eight that can use PE as a carbon source. After 40 days of incubation with Bacillus cereus, the weight loss of PE was 1.6%, while polyethylene terephthalate (PET) and polystyrene (PS) experienced weight losses of 6.6% and 7.4%, respectively. At the same time, *Bacillus gottheilii* had a weight loss rate of 6.2% for PE MPs, 3.0% for PET MPs, 3.6% for PP MPs, and 5.8% for PS MPs. Compared to Auta et al. [130], Tiwari et al. [129] screened bacteria with a higher degradation efficiency, and the body weight of PEMP was reduced by 19.80% after 35 days of *B. brevis* biodegradation. At the same time, there are also many studies that have found that fungi seem to be more efficient in degrading MPs. For example, Soleimani et al. [134] screened 17 strains of LDPE-degrading actinomycetes, including *Streptomyces*, *Nocardia*, and *Rhodococcus*, from the soil of a municipal landfill containing plastic waste. Of these, *Streptomyces* had the best degradation effect. *Streptomyces* caused the LDPE membrane to decrease in weight by 1.58 mg/g/day after 60 days of incubation. Pathak and Navneet [132] isolated five species of bacteria from soil samples that were able to effectively degrade LDPE. After 4 months of incubation, *P. aeruginosa* V1 had the highest degradation rate, and the weight loss rate of LDPE reached 18.21%. Muhonja et al. [133] isolated bacteria and fungi capable of degrading LDPE from soil containing plastic waste in Dandolafu. After 16 weeks of culture, the degradation efficiency of *Bacillus cereus strain* A5,a displayed an average rate of 35.72% ± 4.01% and *Brevibacillus borstelensis strain* B2,2 an average rate of 20.28% ± 2.30% on a 30-micron PE sheet. In addition, Sangale et al. [131] screened two of the best PE-degrading fungal strains, *Aspergillus terreus* MANGF1/WL (pH = 7) and *Aspergillus terreus* PNPF15/TS (pH = 3.5), from 109 strains of fungi isolated from the inter-root soils of mangrove plastic-waste dumping sites on the west coast of India. They observed the changes in weight and tensile strength of PE under different pH conditions. *Aspergillus terreus* MANGF1/WL reduced the weight of the PE strip by 58.51% ± 8.14%, and *Aspergillus sydowii* PNPF15/TS reduced the tensile strength of the PE by 94.44% ± 2.40% within 60 days. From the above results, it can be seen that the degradation effect of fungi is generally higher than that of bacteria.

The insect gut is also an exceptional source of screening for MP-degrading bacteria. Kang et al. [135] isolated bacteria that were able to break down PS in the intestines and feces of mealworms fed PS in an anaerobic environment. Among them, *Ehommaechei* LG3 demonstrated the greatest anaerobic PS-degradation capacity, reducing the weight of the PS membrane by 2.76% ± 0.22% in 15 days. Additionally, it has the capability to break down the PS membrane in an aerobic environment. The weight of the PS film was reduced by 1.94% ± 0.18%. Zhang et al. [137] isolated *Aspergillus flavus* PEDX3 from the gut of the wax moth. After co-culturing with HDPEMPs for 28 days, the molecular mass of HDPEMPs decreased by 3.9025% ± 1.18%. Meanwhile, the appearance of new functional groups was detected by FTIR, confirming the degradation of PE. Yang et al. [136] isolated two strains of bacteria, *Bacillus* sp. YP1 and *Enterobacter asburiae* YT1, from the gut of Indian meal moth larvae. During the 60-day culture period, the PE film was degraded by approximately 10.7% ± 0.2% and 6.1% ± 0.3% for the two strains of bacteria, respectively. From the above findings, we found that the degradation efficiency of these strains on MPs was generally low. This could be attributed to the fact that the ability of insects to degrade MPs is heavily reliant on the microorganisms present in their gut. However, the content of MPs in the insect gut is low compared to that in the natural environment and exists for a shorter period of time. Therefore, it may be difficult to screen for strains with the ability to efficiently degrade MPs.

Natural selection is a simple principle that is easy to implement and can effectively avoid the occurrence of strain degradation while increasing production. However, due to the existence of repair mechanisms such as photorepair, recombination repair, and cleavage repair in microorganisms, as well as the correction function of DNA polymerase, the chance of natural mutations in microorganisms is very low. As a result, natural selection is time-consuming and has a large workload. The effect usually has difficulty meeting the actual demand [144]. In addition, we found that many of the above studies on screening bacteria that degrade MPs through natural breeding methods typically isolated dozens or even hundreds of strains to identify those with the ability to degrade MPs. In future studies, we believe that strains with the ability to degrade MPs can be screened directly by using a culture medium with MPs as the sole carbon source (e.g., adding MPs to a minimal salt medium). This is because only strains with MP-degradation ability can utilize the carbon source in MPs to sustain their growth [118]. This eliminates the need for the step of isolating strains, effectively improving the screening efficiency of natural breeding.

### 4.2. Genetic Engineering Breeding

Genetic engineering is the manipulation of the genome of an organism through biotechnology or modern molecular techniques [145]. Genetic engineering technology involves constructing engineered bacteria at the genetic level to selectively introduce the necessary genetic information into microorganism cells, thereby achieving the targeted breeding of microorganisms. Genetic engineering technology offers greater potential for microbial breeding because it can change the specific sequence of DNA in microorganisms. By precisely manipulating certain sequences in the genome, it is possible to insert, delete, or replace nucleotides in specific genes or sequences of microorganisms [146]. Two methods are typically used in genetic engineering breeding techniques: one is to introduce genes or sequences into microbial cells to obtain the desired expression characteristics; the other is to perform targeted mutagenesis, which replaces the receptors in a specific, effectively mutated gene region. Wild-type copies of bacterial genes can be used to improve certain traits in the desired strain.

**Figure 5:**
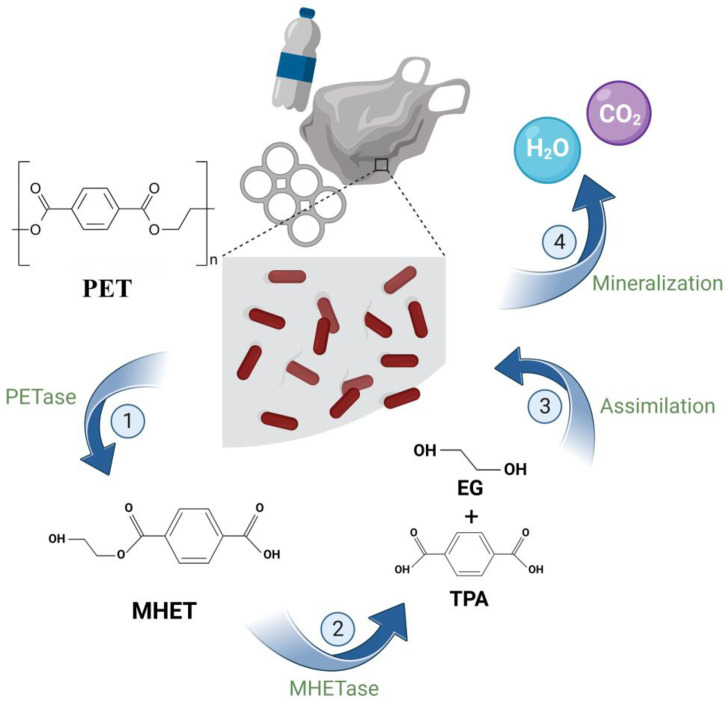
Microbial degradation mechanism of PET. The blue arrow indicates the process of the microbial degradation of PET. Created with BioRender.com.

The primary objective of genetic engineering is to identify, modify, and replicate the genes that are involved in the breakdown process. Achieving this goal is possible through the use of techniques such as antisense RNA technology, PCR, site-directed mutagenesis, and by incorporating suitable hosts, such as *Escherichia coli* [147]. Yoon et al. [139] reported that *Pseudomonas* sp. E4 has the ability to degrade PE. Through the expression of its alkane hydroxylase gene in *Escherichia coli*, the host cells can obtain low-molecular-weight PE mineralization activity. Finally, recombinant *Escherichia coli* mineralized 19.3% of the carbon to CO_2_ over a period of 80 days. Bollinger et al. [140] cloned the gene for the production of a polyesterhydrolase (a PET hydrolase) from *Pseudomonas aestuansigri*. They introduced this gene into *E. coli* to produce the enzyme, but when the temperature was set to 30 °C, it was only capable of breaking down amorphous PET film and not the PET film of commercial PET bottles. However, a variant PE-H (Y250S) was created through site-directed mutagenesis, which had increased activity and was capable of hydrolyzing the PET film from commercial PET bottles. In addition, Ribitsch et al. [141] found that the cutinase produced by *Thermobifida cellulosilytica* (Thc_Cut1) had the ability to degrade PET. They observed whether the degradation rate of cutinase produced by fusion with the hydrophobic enzyme was improved. After fusion, the hydrolysis rate of PET increased by over sixteen times. Furthermore, Austin et al. [142] obtained a PETase (a PET-degrading enzyme) from *Ideonella sakaiensis* 201-F6. After protein engineering, the degradation performance of PET was improved. Ma et al. [143] also performed the protein recombination of PETase obtained from *Ideonella sakaiensis*. Their approach differed in that they placed their focus on the six key residues near the substrate-binding groove, aiming to create PETase mutants using protein engineering to enhance the enzyme’s hydrolysis efficiency. The weight loss rate of the PET film for the mutant with the highest activity was 22.5 mg per μmol L^−1^ PETase per day. Currently, many studies have been conducted on the use of genetic engineering techniques to enhance the degradation ability of PET-degrading bacteria. Figure 5 describes the mechanism by which microorganisms degrade PET.

Although genetic engineering breeding technology can significantly improve the degradation efficiency of MP-degrading bacteria, it has only been studied under laboratory conditions. There is no example of a practical application, so the efficiency is not guaranteed in a real environment. At the same time, the technology also poses environmental risks and raises concerns about ecological safety. The application of genetic engineering technology may have impacts on biodiversity conservation, such as gene pollution and gene loss. Moreover, certain legal policies have restricted the use of genetically modified microorganisms in real environments. These issues create some obstacles for the practical application of this technology in the governance of MPs.

### 4.3. Mutation Breeding

Microbial mutation breeding uses physical or chemical factors to alter the genetic material of microorganisms and induce mutations in microbial genes through artificial means. This process aims to modify the genetic structure and function of microorganisms and identify mutant strains with desirable traits from a pool of mutants. Mutations are rare, reversible, and recessive phenomena, and they are the primary cause of all genetic diversity in any organism [148]. Mutation breeding is a simple, rapid, selective, and versatile technical method. It is also the most commonly used breeding method with the highest success rate in strain cultivation [149]. However, there are only a few applications of this method in the selection and breeding of MP-degrading bacteria. Therefore, in this section, we briefly describe mutagenesis techniques that have potential in the selection and breeding of MP-degrading bacteria.

#### 4.3.1. UV Mutagenesis

UV mutagenesis was one of the earliest methods of biological mutagenesis. The UV spectrum is the same as the absorption spectrum of nucleic acids in cells, with the absorbance of DNA at a wavelength of 250 nm [150]. After the purines and pyrimidines of the DNA and RNA of the strains absorb UV light, the DNA molecule will form a pyrimidine dimer [151], causing deformation of the double-stranded structure. This affects the normal complementary pairing of bases, leading to errors in DNA replication and transcription, resulting in mutations or death. In addition, the formation of dimers can prevent the unwinding of the double strand, thus affecting DNA replication and transcription. UV mutagenesis can cause base conversions, transversions, frameshift mutations, or deletions in bacteria, thereby inducing mutagenesis in them. In the application of this strain-breeding technology, Watanabe et al. [138] obtained a mutant strain of *C. flavus* GB-1 DMC1, which has the ability to degrade biodegradable plastics, through UV mutagenesis. The degradation ability of the mutant strain was more than 2.5-times higher than that of the parental strain GB-1. UV mutagenesis also has some drawbacks, such as its tendency to cause photoreactivation and genetic instability, so the method is not commonly used.

#### 4.3.2. Laser Radiation Mutagenesis

As a new type of mutagen, lasers have the characteristics of high energy density, relative concentration, monochromaticity, and good directionality. Genetic mutations can occur after mutagenesis. A certain amount of laser light irradiates organisms, and the energy can be directly or indirectly deposited onto DNA, causing the molecules to stimulate photodissociation, decomposition, and the free radical reactions of biomacromolecules, resulting in distortions of DNA molecules or chromosomes and accelerating the development of mutant traits. By utilizing lasers to apply heat, light, pressure, and electromagnetic fields, the body’s DNA, RNA, and proteins can be stimulated. This leads to the creation of different substances [152]. This technique has been used in the mutagenesis breeding of microorganisms [153,154,155]. Lotfabad et al. [156] found that the mutant strain MR01-C, which was isolated by the γ-irradiation of MR01 (an indigenous strain of *Pseudomonas aeruginosa*), could increase the production and activity of rhamnolipids. The presence of rhamnolipids can enhance the degradation efficiency of PS [157]. Therefore, this mutagenesis method can be used to select strains with MP-degradation ability.

#### 4.3.3. Microwave Mutagenesis

The equipment needed for microwave mutagenesis is simple. The method is convenient, safe to operate, and does not produce toxic substances. It also allows for the quick generation of a large number of mutants [158]. This method overcomes the shortcomings of UV mutagenesis, such as the ease of photorepair and the high toxicity of chemical mutagenesis, and, at low cost, it is suitable for extensive use in the field of microbial breeding. Microwaves are high-frequency electromagnetic waves. The mutagenic principle is to stimulate the rapid vibration of polar molecules (such as water, proteins, nucleic acids, fats, and carbohydrates). Microwave radiation can induce strong electric polarity oscillations in polar molecules around the cell wall [159,160], causing strong friction between DNA molecules, destroying the hydrogen bonds of DNA molecules and the chemical forces of base accumulation, changing the structure of DNA, and damaging the cell surface of microorganisms [158], resulting in chromosomal changes and producing mutants with variations in desirable genetic traits. The experimental conditions of microwave mutagenesis are not only easy to realize but also overcome the shortcomings existing in UV mutagenesis. However, this technology has not been applied to the selection and breeding of MP-degrading bacteria. Therefore, it can also be applied in the mutagenesis selection of strains that degrade MPs.

#### 4.3.4. ARTP (Atmospheric Room Temperature Plasma) Mutagenesis

The ARTP breeding technique is an emerging mutagenesis technique. The plasma generated by ARTP is rich in various chemically active particles. These active particles can exert multiple effects on cells, such as damaging genetic material and causing structural changes in the cell membrane, as well as permeating proteins [161]. When particles act on DNA, they can cause damage to its structure. As a result, cells will initiate the SOS repair mechanism to form stable genetic mutations [162]. ARTP mutagenesis breeding is characterized by a low cost, easy operation, a high mutation rate, and genetic stability [163]. It has been extensively utilized in the mutagenesis of bacteria, fungi, and microalgae [164]. Currently, no research has been conducted on using ARTP mutagenesis to obtain strains that can improve the efficiency of degrading MPs in bacteria breeding. This is also a breeding method worth considering for researchers to improve the efficiency of MP degradation.

In conclusion, gene mutation is the primary cause of microbial variation, and artificial mutagenesis is an important means to accelerate gene mutations. Breeding technology based on artificial mutagenesis has the advantage of being fast, high-yielding, and simple. Therefore, it is an important method for microbial breeding. However, artificial mutagenesis is very random, and therefore, it must be combined with large-scale screening to obtain good results. If the screening method is appropriate, mutated strains can be obtained in a targeted manner. In addition, negative mutants should be eliminated, and positive mutants with excellent characteristics should be screened out in order to achieve the purpose of breeding good strains.

## 5. Perspectives

Currently, researchers have made some progress in the breeding of MP-degrading bacteria. More and more efficient degrading strains have been discovered and characterized, providing new solutions for managing MP pollution [165]. Researchers have improved the degradation performance and production efficiency of strains by continuously optimizing the technical methods of strain breeding. However, current research still faces some challenges. First, in the current breeding of MP-degrading bacteria, natural selection and genetic engineering techniques are still the most commonly applied methods. However, the process of natural selection and breeding is long and tedious. Genetic engineering technology also has the potential problem of genetic contamination and biosafety risks. Although there are many methods of strain breeding, mutagenic breeding technology is classical and reliable. It will not pose any risks to biological and environmental safety issues. It is an important method of microbial breeding, but it is not widely used in the mutagenic breeding of MP-degrading bacteria. Therefore, in the subsequent breeding of MP-degrading bacteria, more consideration can be given to using mutagenesis to obtain strains that can efficiently degrade MPs. Secondly, in practical applications, how to achieve the efficient production and large-scale application of strains still needs to be further explored [166].

In the future, with the continuous progress of science and technology and in-depth research, strain-breeding technology will play a more important role in the breeding of MP-degrading bacteria. Secondly, the degradation performance and production efficiency of strains are expected to be further improved by optimizing mutation breeding methods. At the same time, relevant government departments can formulate corresponding laws and regulations regarding the use of genetic engineering breeding technology. This will allow for practical application, rather than just remaining in the laboratory stage. In addition, the use of synthetic biology technology to build microbial cell factories with multiple functions to achieve the efficient degradation and resource utilization of MPs is also a future research focus. In future research, we should pay attention to the study of degradation efficiency and degradation mechanisms in biodegradation methods.

## 6. Conclusions

In this paper, the sources of MPs in soil were analyzed, and the severity of the pollution caused by MPs was clarified. At the same time, this paper summarizes, for the first time, the strain-breeding technology with the ability to enhance the degradation efficiency of MP-degrading bacteria. This aims to overcome the problem of the low efficiency of MP-degrading bacteria and provide a new direction for cultivating efficient MP-degrading bacteria in the future. Overall, unpretreated PE is the most difficult plastic to biodegrade, while PS with more functional groups is more readily biodegradable. For microorganisms with the ability to degrade plastics, the degradation efficiency of fungi is usually higher than that of bacteria. In addition, the mutagenic breeding technique deserves more consideration for use in subsequent studies. This paper provides a valuable reference for the selection and breeding of efficient MP-degrading bacteria.

## Figures and Tables

**Table 1:** The abundance and distribution characteristics of MPs in soils in some typical areas.

Country or Region	Soil Type	Particle Size (mm)	Sampling Depth (cm)	Polymer Type	Form	Abundance	Reference
Australia	Industrial soil	0.05–1	—	PE, PVC	—	300–67,500 mg kg^−1^	[101]
Switzerland	Floodplain soil	<2	0–5	PE, PS, PVC	particle	55.5 mg kg^−1^	[102]
Peking	Vegetable field	<5	0–20	PE, PP	debris, fiber, film, particle, foam	160–5220 items kg^−1^	[103]
Mexico	Vegetable field	<5	0–20	PE	particle	870 ± 190 particles kg^−1^	[104]
Germany	Cropland	1–5	0–5	PE, PP, PS	fragment, fiber, film	0–1.25 MPPs per kilogram	[53]
Canada	Cropland	0.11	0–15	PE, PP, PET	fiber, fragment	18–541 MP kg^−1^	[75]
Iran	Cropland	0.04–0.74	0–10	—	debris, fiber	67–400 particles kg^−1^	[105]
Spain	Cropland	<5	0–10	PE	particle	2000 particles kg^−1^	[106]
Pakistan	Cropland	0.05–5	0–10	—	debris, fiber, film, particle, foam	2200–6875 MPs kg^−1^	[107]
Chile	Cropland	<2	0–25	PES, PVC	fiber, film	600–10,400 particles g^−1^	[108]
Denmark	Cropland	0.02–0.5	0–10	PE, PP, PA	—	82,000–236,000 no. kg^−1^	[109]
Yunnan	Cropland	0.05–1	0–10	PE	fragment, fiber, film	7100–42,960 particles kg^−1^	[110]
Korea	Cropland	0.1–2	0–5	PE, PS, PP, PET	fiber, fragment, film	10–7630 items kg^−1^	[111]
Wuhan	Cropland	0.02–5	0–5	PA, PP	fiber, fragment, particle, foam	320–12,560 items kg^−1^	[112]
Shaanxi	Cropland	<0.49	0–10	PE, PP, PVC, PS, PET	fiber, fragment, film, particle	1430–3410 items kg^−1^	[113]
Xinjiang	Cropland	0.15–2	0–0.2	PE	fiber, fragment	91.2–308.5 mg kg^−1^	[51]

**Table 2:** MP-degrading bacteria were obtained by different strain-breeding techniques.

Plastic Types	Microorganism	Environment	Sampling Site	Breeding Method	Degradation Time (Days)	Weight Loss (%)	Reference
PP	* Exiguobacterium marinum * a-1	Marine	Bohai Bay	Natural selection	80	9.20	[125]
PE PP	*Bacillus paramycoides* *Bacillus cereus*	River	Vaigai River, Madurai, India	Natural selection	21	78.99 ± 0.005 63.08 ± 0.009	[126]
PE	*Kocuria palustris* M16 *Bacillus pumilus* M27 *Bacillus subtilis* H1584	Marine	Arabian sea coast , India	Natural selection	30	1 ± 0.033 1.5 ± 0.038 1.75 ± 0.06	[127]
PET	*Vibrio* sp. PD6 *Aspergillus* sp.	Marine	Bay of Bengal, Sunderban, West Bengal	Natural selection	42	35 22	[128]
PE	*B. brevis*	Land	Dehradun and Haridwar in Uttarakhand in India	Natural selection	35	19.8	[129]
PE PET PP PS	*Bacillus cereus* *Bacillus gottheilii*	Land	Mangrove ecosystems in Peninsular Malaysia	Natural selection	40	1.6, 6.6, —, 7.4 6.2, 3.0, 3.6, 5.8	[130]
PE	*Aspergillus terreus* strain MANGF1/WL *Aspergillus sydowii* strain PNPF15/TS	Land	Growing mangroves surrounded by marine water along the west coast of India	Natural selection	60	58.51 ± 8.14 94.44 ± 2.40	[131]
LDPE	*P. aeruginosa* V1 *B. subtilis* V8 *P. aminophilus* B1 4- *P. putida* C 2 5 *A. calcoaceticus* V4	Land	Haldwani, Bhimtal, and Rishikesh soil samples	Natural selection	120	18.21 16.12 11.72 13.30 15.44	[132]
LDPE	*Bacillus cereus* strain A5,a *Brevibacillus borstelensis* strain B2,2	Land	Dandora dumpsite	Natural selection	112	35.72 ± 4.01 20.28 ± 2.30	[133]
LDPE	*Streptomyces alborgiseolus IR-SGS-T10* *Nocardia* sp. *IR-SGS-T3* *Rhodococcus ruber IR-SGS-T6*	Land	Yazd city Kelardasht forest Tehran city	Natural selection	60	9.5 ± 0.3 5.98 ± 0.72 6.23 ± 0.5	[134]
PS	*Ehommaechei LG3*	Insect	The gut of Tenebrio molitor larvae	Natural selection	15	2.76 ± 0.22	[135]
LDPE	*Bacillus* sp. *YP1* *Enterobacter asburiae YT1*	Insect	The gut of waxworms	Natural selection	60	10.7 ± 0.2 6.1 ± 0.3	[136]
HDPE MPP	*Aspergillus flavus PEDX3*	Insect	The gut of wax moth *G. mellonella* larvae	Natural selection	28	3.9025 ± 1.18	[137]
BP	*C. flavus GB-1 DMC1*	Plant	Rice husks	Mutagenesis breeding	—	—	[138]
LMWPE	*Pseudomonas* sp. *E4*	Marine	Manripo Beach, Yellow Sea, Korea	Genetic engineering breeding	80	19.3	[139]
PET	*Pseudomonas aestusnigri*	Marine	—	Genetic engineering breeding	—	—	[140]
PET	*Thermobifida cellulosilytica*	—	—	Genetic engineering breeding	—	—	[141]
PET	*Ideonella sakaiensis 201-F6*	—	—	Genetic engineering breeding	4	—	[142]
PET	*Ideonella sakaiensis*	—	—	Genetic engineering breeding	2	22.5 mg per μmol L^−1^ PETase per day	[143]

HDPE MPP: High-density polyethylene microplastic particle. BP: Biodegradable plastic. LMWPE: Low-molecular-weight polyethylene.

## Data Availability

All data analyzed during this study are included in this article.
